# MEDIATING EFFECT OF GRIP STRENGTH TRAJECTORY ON THE ASSOCIATION BETWEEN EXERCISE AND WEAKNESS: A DYADIC APPROACH

**DOI:** 10.1093/geroni/igz038.1074

**Published:** 2019-11-08

**Authors:** Susanna Joo, Hye Won Chai, Hey Jung Jun

**Affiliations:** 1 Yonsei University, Seoul, Korea, Republic of; 2 The Pennsylvania State University, University Park, Pennsylvania, United States

## Abstract

This study aimed to explore the mediating effect of grip strength trajectory on the longitudinal association between regular exercise and weakness in grip strength using a dyadic approach. We used six waves of the Korean Longitudinal Study of Aging (KLoSA) collected every two years from 2006 to 2016. The sample was middle and old-aged Korean couples who participated in all six waves of the survey (N=1,967). The outcome variables were husbands’ and wives’ grip strength at Wave 6, coded as a binary variable (0=clinically weak, 1=normal). The mediating variables were husbands’ and wives’ trajectories of grip strength across Waves 1 and 5. Independent variables were three dummy variables indicating couple’s participation in regular exercise (both engaged in regular exercise, only husband did, only wife did). Reference group was both not doing regular exercise. Results showed several significant mediational pathways. For husbands, engagement in regular exercise of both spouses and only husband were associated with higher grip strength at Wave 1, and slower decline in grip across waves was related to lower likelihood of having a clinically weak grip strength at Wave 6. As for wives, engagement in exercise of both spouses was associated with higher grip strength at Wave 1 which in turn was related to lower likelihood of having a clinically weak grip strength at Wave 6. These results suggest longitudinal dyadic processes through which engagement in regular exercise affects weakness in grip strength among older Korean couples.

